# Characterization and Energy Densification of Mayhaw Jelly Production Wastes Using Hydrothermal Carbonization

**DOI:** 10.17113/ftb.61.01.23.7783

**Published:** 2023-03

**Authors:** Viral Sagar, MeiLan Hardin, Narendra Kumar, Joan G. Lynam

**Affiliations:** Department of Chemical Engineering, Louisiana Tech University, P.O. Box 10348, 600 Dan Reneau Drive, Ruston, LA 71272, USA

**Keywords:** berries, food, pomace, biofuel, FTIR, HTC

## Abstract

**Research background:**

Mayhaw jelly, made from mayhaw berries from the southern United States, is a popular food product that on processing produces a berry pomace waste. Little information is available in the literature about this waste or how to valorize it. This study investigated this food production waste and its possibilities for conversion to a biofuel.

**Experimental approach:**

Dried mayhaw berry wastes were characterized with fiber analysis using the US National Renewable Energy Laboratory methods. After drying and grinding, hydrothermal carbonization was applied to the mayhaw berry wastes, the mayhaw waste without seeds, and mayhaw waste seeds. Fourier transform infrared spectroscopy (FTIR) was performed on mayhaw berry wastes, mayhaw waste without seeds, and mayhaw waste seeds. Calorimetry revealed the fuel value of each component of the waste and of the dried mayhaw berry wastes without any component separated. Friability testing on pellets of the biomass investigated their durability.

**Results and conclusions:**

Fiber analysis indicated a high proportion of lignin compared to cellulose in the dried mayhaw waste. Hydrothermal carbonization did not enhance the fuel value of the seeds due to their tough outer coat that inhibited hydrothermal carbonization’s high ionic-product water penetration. Other mayhaw berry waste samples had enhanced fuel value after treatment at 180 or 250 °C for 5 min, with a higher fuel value attained for 250 °C treatment. After hydrothermal carbonization, the wastes were easily pelletized into durable pellets. Fourier transform infrared spectroscopy characterization indicated raw seeds had high lignin content, as did the hydrothermal carbonization-treated mayhaw berry wastes.

**Novelty and scientific contribution:**

Hydrothermal carbonization is a process not previously applied to mayhaw berry wastes. This study fills in the gaps of this waste biomass’ potential to become a biofuel.

## INTRODUCTION

Converting biomass to biofuels or bio-products is essential in developing a sustainable, non-fossil fuel-based economy. If waste biomass from food production is used, the feedstock will be inexpensive and in a central location rather than in a field. Recently, there has been a growing interest in the use of berry waste due to its high nutritional value, various health benefits, low cost, and eco-friendly nature (*1*). Berry waste is most often produced as a by-product from the pressing process to produce jams, jellies, juices, and wines (*1*). Berry waste or berry pomace consists of seeds, peeled skin, and pulp that make up approx. 20% of the whole berry (*2*). It has normally been composted but microbiological quality and safety have been concerns with this utilization method for berry waste (*3*). Therefore, other safer and more effective methods of utilizing berry waste have been investigated and implemented in different industries.

Berry seed oil is useful in the cosmetics industry due to their fatty acid compositions, high tocopherol content, and resistance to oxidative stress resulting in better shelf life (*1*, *4*). The seed oil is utilized in toothpaste, shampoo, lipstick, skin oils/creams, and various other cosmetic products (*1*, *4*). Nevertheless, methods to extract berry seed oil require an extraction process that uses hazardous chemicals such as hexane (*4*, *5*).

Various methods of extraction and modifications to the berry waste have been investigated to determine the best method to maintain or enhance the waste’s nutritional value. One such study investigated how fermentation affected the nutritional content of blueberry pomace. Results showed that fermentation enhanced antioxidative properties, microbiota community structure, and increased phenolic compound content and the production of short-chain fatty acids (*e.g.* acetic, butyric, and lactic acids) (*6*). Fermentation *via* controlled pH in the acidic range also provided a suitable method to obtain bioactive compounds from strawberry waste (*7*). However, fermentation is a long and complicated process. In another study, berry pomace was complexed with rice-pea protein isolate blends to create a protein polyphenol aggregate particle that was then analyzed in an *in-vitro* gastrointestinal model (*8*). The modified particles contained higher polyphenol concentrations, retained higher antioxidant/anti-inflammatory activity, and were more stable than the unmodified samples (*8*). High polyphenolic content has also been reported after acid hydrolysis of strawberry, raspberry, blueberry, and blackberry decoctions (*9*). Researchers have also explored various solvent extraction methods, such as comparing supercritical CO_2_ solvents to conventional solvents in lingonberry pomace (*10*). Supercritical CO_2_ extraction did not perform as well as conventional extraction in terms of radical scavenging (*11*). Kitrytė *et al.* (*12*) investigated enzyme-assisted pressurized extraction applied to chokeberry pomace and were able to extract a number of antioxidants, as well as monosaccharides (*12*).

If the wastes from processing are considered, blueberry pomace has higher total phenolic and total anthocyanin content than raspberry pomace (*13*). Furthermore, blueberry pomace has higher protein content than cranberries (*14*). Blueberries and raspberries both have a good balance of n-6 and n-3 fatty acids and high beta-sitosterol content, thus making them reliable sources of polyunsaturated fatty acids (PUFAs) and phytosterols (*15*). Raspberry seeds contain a higher percentage of oil, which is a reason why it is so widely utilized in cosmetics and even some pharmaceuticals (*1*). These differences in berries can affect how much a certain type of berry waste is used and can determine the industries in which they can be applied. One such usage of berry waste that has not been completely investigated yet is the use of berry waste as a biofuel. This may be because different types of berries have a high degree of variability, as do their individual berry waste components, which hinders their efficient application in energy production (*16*). Little research is available on this topic in the literature.

The present study investigates the conversion of mayhaw berry wastes to a pelletizable biofuel. The method used was hydrothermal carbonization (HTC), a process not previously applied to mayhaw berry wastes. The HTC process converts raw biomass into a coal-like product called hydrochar through the temperature and pressure conditions resembling the coalification process in geological rocks (*17*). Compared to the moist biomass, hydrochar has a higher fuel value and available carbon content. The HTC process is also known as wet torrefaction to some waste to energy experts. An advantage of using the HTC process on biomass is that only water, with no hazardous chemicals, is added to the biomass (*18*). This means that wet biomass directly sourced from a jelly-making process could undergo HTC with no energy-intensive drying needed.

## MATERIALS AND METHODS

### Chemicals and materials

Sulfuric acid (reagent grade, 95–98%) was purchased from Millipore Sigma, Merck (St. Louis, MO, USA). Denatured ethanol (90.5%) was purchased from Duda Energy (Decatur, AL, USA). Nylon membrane discs of pore size 0.45 µm were bought from Foxx Life Sciences (Salem, NH, USA).

**Fig. S1:**
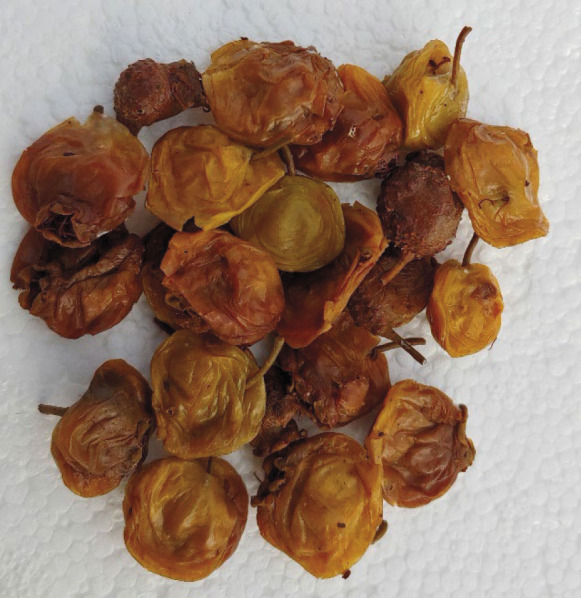
Dried mayhaw berry waste as received

Raw mayhaw berry wastes were acquired from Mr. Michael Book of Mayhaw Market, a local farmer in Ruston, LA, USA as shown in Fig. S1. The dried mayhaw berry waste used for the purpose of this research study was separated from any remaining leaves after the jelly making process.

### Microscale preparation and characterization of mayhaw berries

Dried mayhaw berry wastes were ground in a tabletop coffee bean grinder to separate the skins of the berries from the seeds for about 30 s. This grinding allowed the separation of the biomass into three categories: mayhaw berry wastes (MB), mayhaw seeds (MS), and mayhaw berry waste without seeds (MH) for further analysis. The ground samples were further hand sieved through a #20 mesh (0.84 mm openings) and a #80 mesh (0.18 mm openings) from Gilson Company Inc. (Lewis Center, OH, USA) for aggregate particle size separation. The pass-through samples from the sieving step were collected separately as #20 pass through (0.18–0.84 mm particle size) and #80 pass through (less than 0.18 mm particle size). The three sample types used in this investigation were mayhaw berry wastes (MB), mayhaw seeds (seeds separated from the berry skins using the grinding and sieving process, MS), of particle size 0.84 to 0.18 mm, and mayhaw berry wastes without seeds (MH). The small quantity of stems was lumped in with the MH portion. The samples were weighed on an analytical balance in triplicate to quantify the fractions of mayhaw seeds and MH.

### Fiber analysis

Fiber of the as received dried mayhaw waste was analyzed using the standard National Renewable Energy Laboratory (NREL) protocols LAP/TP-510-426 18 through 22 (*19*). Soluble components within the biomass, consisting of non-structural parts, must be removed from the biomass before compositional analysis. This is performed to prevent any discrepancies in later analytical procedures as per NREL protocols. Biomass extractives capable of dissolving in water and/or ethanol solvent were removed by Soxhlet extraction. Biomass (~8 g) was placed in a thimble and 200 mL of ethyl alcohol were transferred into a conical flask. The thimble with biomass was inserted carefully into a Soxhlet siphon tube and kept above the conical flask with ethyl alcohol. The whole setup was kept in an oil bath at 80 °C for 24 h, and after that the biomass from the thimble was taken out and its loss was measured. The percentage of the removed extractives was then calculated.

The cellulose, hemicellulose, and lignin content of the samples were determined by quantitative saccharification with acid hydrolysis and subsequent HPLC analysis, using NREL protocols LAP/TP-510-426 18 through 22 (*19*). For the dried mayhaw waste biomass, ethanol extraction was carried out to remove the non-structural components of the biomass (as described in the previous section) prior to acid hydrolysis and thus the biomass fraction regarded as extractives was removed from the whole biomass sample. The concentrations of glucose, xylose, arabinose, galactose, and mannose were quantified using an HPLC (ThermoFisher Scientific, Waltham, MA, USA) equipped with refractive index detector and an Aminex HPX-87P column (300 mm×7.8 mm; Bio-Rad Laboratories, Inc., Hercules, CA, USA). The column temperature was maintained at 80 *°C* and the flow rate was 0.6 mL/min of deionized (DI) water. Each experiment was performed in triplicate. Total cellulose release upon acid hydrolysis was determined as the sum of cellobiose and glucose and the total hemicellulose release was determined as the sum of xylose, galactose, arabinose, and mannose. The sum of acid-insoluble and acid-soluble lignin was represented as total lignin content available in each sample.

The NREL fiber analysis procedure was followed to measure the acid-soluble lignin and acid-insoluble lignin from the biomass with acid hydrolysis (*19*). A UV-2401PC spectrophotometer (Shimadzu Corporation, Kyoto, Japan) was used to analyze the acid-soluble lignin in the biomass. Once the biomass extractives were removed, samples were hydrolyzed with 72% H_2_SO_4_ and, with the hydrolysis, acid-soluble lignin in the sample was dissolved. Subsequently, 3 mL of this sample were measured and diluted 10 times with DI water. With DI water as a blank, analytes (lignin) in the sample were measured by absorbance at a wavelength of 260 nm. To determine the acid-insoluble lignin, as per the NREL procedure, the solid remaining after acid solid lignin was dissolved was washed, dried, and then held at 750 °C for 3 h. By mass difference, acid-insoluble lignin was found.

As per NREL/TP-510-42622, mayhaw waste samples of approx. 0.5 g in triplicate were heated in crucibles to 575 °C in a furnace and held for 24 h (*20*). The mass fraction (in %) remaining was considered as ash (inorganics).

### Hydrothermal carbonization

The various types of mayhaw biomass samples were hydrothermally carbonized in a Parr reactor (Parr Instrument Company, Moline, IL, USA). Hydrothermal carbonization (HTC) was used to study the effects of high temperature and pressure on the berry wastes and to drive the lignin separation within it. In these experiments, the DI water to biomass mass ratio was 10:1.

After HTC treatments at 180 or 250 °C for 5 min, the mayhaw biomass and the liquid with it were brought to room temperature through quenching. Once the HTC-treated biomass was cooled, solid biomass was separated from the solution using a nylon filter (0.45 µm pore size) membrane. A simple filtration unit was set up by connecting a Buchner funnel with a filter and a filtration flask to a vacuum pump inside the fume hood. The HTC-treated biomass with the solvent was filtered and the filter cake (filtride) from the filtration was separated using vacuum. Filtered biomass was dried at 105 °C for 24 h prior to weighing, to ensure all moisture was thoroughly removed.

### Bomb calorimeter

Higher heating values (HHV) of combustion for samples were measured in an adiabatic oxygen bomb calorimeter (1341EB bomb calorimeter; Parr Instrument Company) fitted with continuous temperature recording. Samples were placed in a drying oven at 105 °C for 24 h prior to analysis, and HHVs are reported on a dry, ash-free basis. Each sample (0.4 to 0.5 g) was weighed into a metal crucible and 10 cm of fuse wire was bent to touch the top of the biomass. Each sample was oxygenated to 3.04 MPa pressure in the vessel. DI water (1 L) was added to the calorimeter. Measurements of initial water temperature, initial crucible mass, initial fuse wire mass, biomass sample mass, final water temperature, crucible mass after ignition, fuse wire mass after ignition, and ash mass were recorded. The standard error in HHV was determined by running the procedure 5 times on mayhaw wastes without seeds. HHV was calculated as follows:


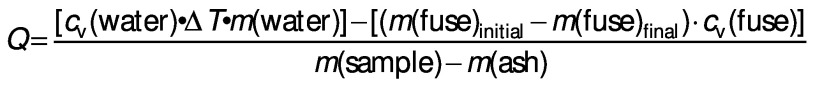
 /1/

where *c*_v_(water)=4.186 J/(kg·K) is the specific heat of water, *c*_v_(fuse)=5.86·10^6^ J/(kg·K) is the specific heat of the fuse material, Δ*T* is the change in temperature in K, *m*(water)=0.001 kg (assuming *ρ*(water)=1000 kg/m^3^ of DI water).

Energy densification was calculated as the heat of combustion of the HTC-treated biomass divided by the heat of combustion of raw dried biomass (no units).

### Pelletization

Mayhaw samples were pelletized to show the effects of compaction of the material for energy densification and to study the pellets for transportation feasibility *via* friability test. Pelletization was carried out in a 13-mm internal diameter cylindrical hardened steel dry pressing die set from Across International (#SDS13.H, Livingston, NJ, USA) heated at 140 °C. Pressure was applied using a crankshaft hydraulic compression machine (model 50H, 50-ton capacity; Dake Corporation, Grand Haven, MI, USA) with 5 ton of pressure and holding this pressure for 30 s. Sample preparation involved taking 1 g biomass and adding 0.2 g DI water to it for wetting purposes. The friability test used a BEXCO Tablet Digital Friability Test Apparatus (single drum) with i-therm KTM-443 Timer (Busan, Korea). Pellets of mayhaw waste (raw and HTC-treated) were loaded in the transparent acrylic drum for 1 and 24 h at a drum rotation speed of (25±1)×*g*. The acrylic drum’s arm carried the pellets along with it up to a predetermined height of (156±2) mm and allowed them to fall from that specified height, while the drum was rotating.

### Fourier transform infrared spectroscopy

A Mattson Genesis II FTIR (Mattson Technology, Fremont, CA, USA) was used to obtain the spectra of the untreated and HTC-pretreated biomass samples. KBr pellets, the standard method to prepare solid samples for Fourier transform infrared spectroscopy (FTIR), were prepared with 1 mg of sample mixed with 100 mg of KBr. The pellets were made with a pellet holder press by applying pressure. Single beam spectra of the samples were collected with 32 scans with resolution 2 cm^−1^ from wavenumber 4000 to 500 cm^−1^. FTIR spectroscopy was performed on the mayhaw seeds (MS) and mayhaw berry wastes without the seeds (MH). FTIR spectroscopy was also performed on mayhaw berry wastes under three conditions: 1) before treatment, 2) after HTC treatment at 180 °C, and 3) after HTC treatment at 250 °C.

### Statistical analysis

Data measurements were made in triplicate and standard error bars are shown in figures, all calculated using Microsoft Excel 2016 (Microsoft Corp., Redmond, WA, USA).

## RESULTS AND DISCUSSION

Dried mayhaw samples were characterized and hydrothermal carbonization (HTC) was performed on the mayhaw seeds (MS) and mayhaw berry waste without the seeds (MH). The focus of the work was on the solid products of the HTC runs, particularly on their potential for use as fuel pellets.

### Mayhaw waste characterization

#### Component fractions

**Fig. S2:**
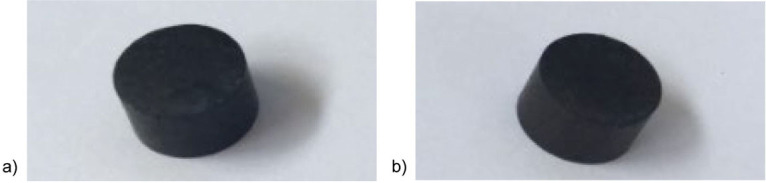
Pellets of: a) HTC-treated at 250 °C dried mayhaw berry waste, and b) HTC-treated at 250 °C mayhaw berry waste without seeds. HTC=hydrothermal carbonization

The mayhaw seeds and mayhaw berry waste without the seeds each comprised about half of the received dried mayhaw waste, as shown in Fig. S2. On average, mayhaw berry waste without the seeds was the smaller portion by mass at (39±4.9) % of the received biomass, and mayhaw seeds averaging (48±6.5) % of the received biomass was the larger portion by mass (data not shown). The remainder of the waste biomass (13%) was unable to be collected because its particle size was too small. Thus, both components were similar in their mass for the waste biomass as received.

#### Mayhaw fiber composition

From the NREL fiber analysis (*19*), dried mayhaw berry wastes were comprised of 26.2% cellulose, 19.4% hemicellulose, 39.6% lignin, 12.0% extractives and 2.7% ash (inorganics). Blueberries as a whole fruit have been found to contain higher amounts of lignin than cellulose (28.4% lignin compared to 16.0% cellulose) (*21*). The ratio of lignin to cellulose in dried mayhaw berry wastes is thus somewhat like that found in blueberries. Little other data can be found on berry skins in the literature.

### Higher heating value

**Fig. 1:**
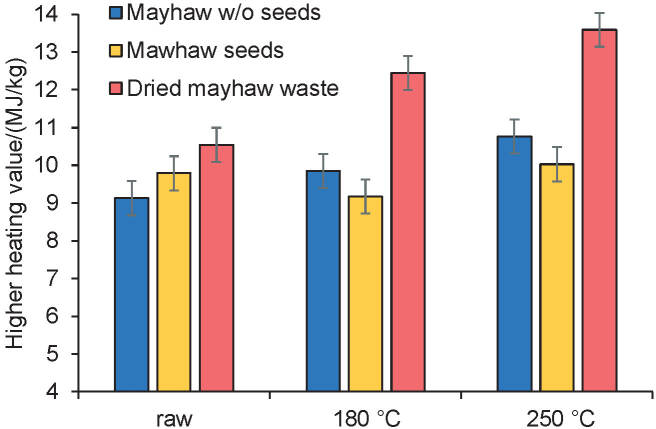
Higher heating value (HHV) of mayhaw berry waste without seeds (MH), mayhaw seeds (MS), and dried mayhaw berry waste as received (MB). HTC=hydrothermal carbonization

As seen in Fig. 1, HTC increased the higher heating value (HHV) of the MH samples and the dried mayhaw berry wastes samples (Eq. 1). The higher HTC temperature of 250 °C (13.6 MJ/kg for dried MB) increased the HHV significantly more than the 180 °C HTC treatment (12.5 MJ/kg for dried MB). With HTC of 250 °C of dried mayhaw berry wastes, energy densification was 1.3, while HTC of 180 °C of dried mayhaw berry wastes gave an energy densification 1.2.

**Fig. 2:**
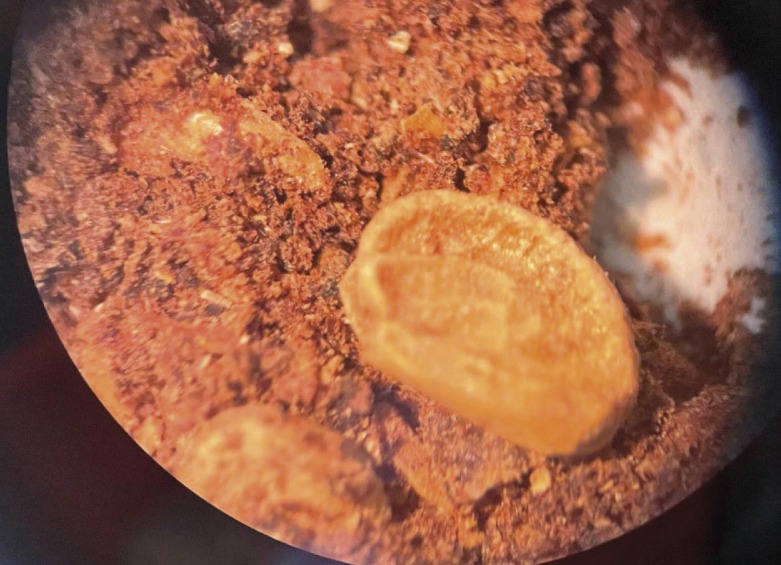
Dried mayhaw waste as seen under an optical microscope with 10× zoom

For mayhaw berry waste without the seeds, energy densification was 1.1 for the 180 °C HTC treatment and 1.2 for the 250 °C HTC treatment. Mayhaw seeds did not appear to be affected by the HTC treatment. Bomb calorimetry of seed samples gave energy densification of 0.94 from 180 °C HTC and 1.0 from 250 °C HTC. This lack of effect from HTC on mayhaw seeds results from the impervious seed coats that protect the seeds, particularly berry seeds, from premature opening (*22*). Huth *et al.* (*23*) have characterized the exterior of the seeds with higher lignin as more resistant to water penetration, which suggests that the brief period of time they underwent HTC was not sufficient to achieve penetration of the seeds. However, this raises the question of why the mayhaw wastes that included seeds showed increased energy densification. An explanation for this phenomenon may be the fact that the seeds that were combined with the remainder of the mayhaw waste in its natural form were ground to pass a 20 mesh (0.84 mm). The seed outer layer was thus disrupted, giving the water under HTC conditions the ability to remove lower fuel value components, leaving higher HHV lignin as part of the solid product. In addition, ground seed particles in the mayhaw waste are likely to have hard irregular edges as seen in Fig. 2. Since stirring occurs during HTC, these edges may abrade the softer components of the mayhaw waste, allowing the water solvent access to more surface area to remove lower HHV components. This could account for the greater energy densification of the dried mayhaw berry wastes.

Mass yields of solid product compared to raw biomass for HTC tended to correspond with HHV increases, with lower mass yields indicating higher HHV. For HTC at 180 °C of mayhaw berry waste without the seeds and mayhaw berry wastes, mass yields were near 63%, while mass yields for 250 °C were lower, near 50%. HTC at 180 °C of mayhaw seeds had a high mass yield of 84%, but a lower mass yield for 250 °C of 54%. The underlying reason for this trend is that HTC removes lower fuel value components such as hemicellulose and cellulose, while retaining the lignin that has a higher fuel value (*24*). In addition, higher HTC process temperature can cause cellulose to condense into structures with more bonds, as well as increasing numbers of double bonds. These condensed structures can have fuel values higher than virgin cellulose as found in raw biomass (*25*). Values for the pH of the liquid that resulted from HTC treatment were approx. 4 for all runs. Typically, acetate groups, attached to xylan or other hemicelluloses in the biomass, detach to form acetic acid during HTC, reducing the resulting liquid’s pH compared to the neutral pH of DI water initially added (*26*).

### Pellet durability

The goal of pelletization is to make biomass fuel pellets more durable, so that they can be transported without loss of mass to produce small, potentially flammable particles (*27*). Pellets of HTC-treated and -untreated mayhaw wastes are shown in Fig. S2. After 1 h of tumbling, the loss was 0.79, 1.97, 2.86, 13.80 and 24.42% for the HTC-treated dried mayhaw berry waste at 250 °C, HTC-treated mayhaw berry waste without seeds at 250 °C, mayhaw seeds, mayhaw berry waste without seeds and MB, respectively (data not shown).

The pellets made from 250 °C HTC-treated biomass appeared to show lower loss of mass after 1 h of tumbling than the untreated biomass of the same type. Mayhaw seeds pellets gave improved durability compared to mayhaw berry waste without seeds pellets that contained the skins. This finding could be attributed to the hard seed coat, which also contains a higher lignin content (*23*). When added to raw biomass, high-lignin HTC solids have been reported to improve pellet durability, thus acting as a binder (*28*). Continuation of tumbling for 24 h indicated that pellets from 250 °C HTC of mayhaw berry wastes showed a loss of 8.36%, while mayhaw berry waste without seeds pellets undergoing the same HTC treatment had a 2.09% loss of mass. Longer tumbling obviously removed more mass, but again the presence of seeds in the pellet may have abraded skin-related structure and allowed loss. For comparison, untreated mayhaw berry waste without seeds that had been pelletized lost 31.86% of its mass after 24 h of tumbling. The HTC process reduced friability and increased durability in the pellets made from the 250 °C HTC solid product.

### FTIR spectroscopy

**Fig. 3:**
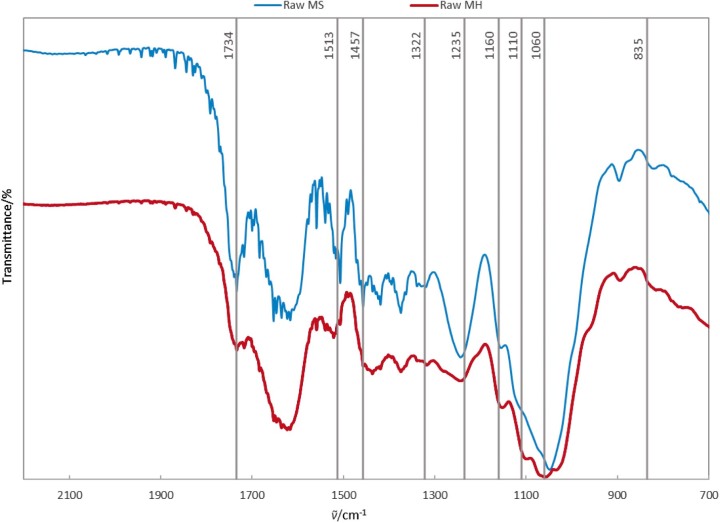
FTIR spectra of mayhaw seeds (MS, top spectrum) and mayhaw berry waste without seeds (MH, bottom spectrum) showing bonds of interest

FTIR can be used to compare the prevalence of biomass components in samples (*29*). FTIR spectra of untreated mayhaw seeds and mayhaw berry waste without seeds in Fig. 3 show bonds of interest.

Several differences in the areas of the spectra vibrations are notable when comparing FTIR spectra of raw mayhaw seeds and raw mayhaw berry waste without seeds. For the MS, more prominent lignin vibrations existed at 835 cm^-1^ (syringyl lignins (*30*, *31*)), 1457 cm^-1^ (CH_2_ deformation/stretching lignin and xylan (*32*)), 1513 cm^-1^ (aromatic skeletal vibration (*33*-*36*)), and 1734 cm^-1^ (ester-linked acetyl, feruloyl and p-coumaroyl groups between hemicellulose and lignin (*37*, *38*)). More prominent cellulose and hemicellulose vibrations were noted in the raw mayhaw berry waste without seeds at 1060 cm^-1^ (C-O stretching vibration (*39*)), 1110 cm^-1^ (C-OH skeletal vibration cellulose and hemicellulose (*40*), and 1160 cm^-1^ (C-O-C asymmetric stretching cellulose I and cellulose II (*39*)). Since lignin vibrations were more predominant in the mayhaw seeds component, and cellulose and hemicellulose appeared larger in the mayhaw berry waste without seeds portion, it is likely that the mayhaw seeds contained more lignin. As discussed above, lignin coating of the seed prevents its premature opening. More cellulose in the mayhaw berry waste without seeds portion may encourage the ingestion by animals and the later expelling of the seed with fertilizing manure.

**Fig. 4:**
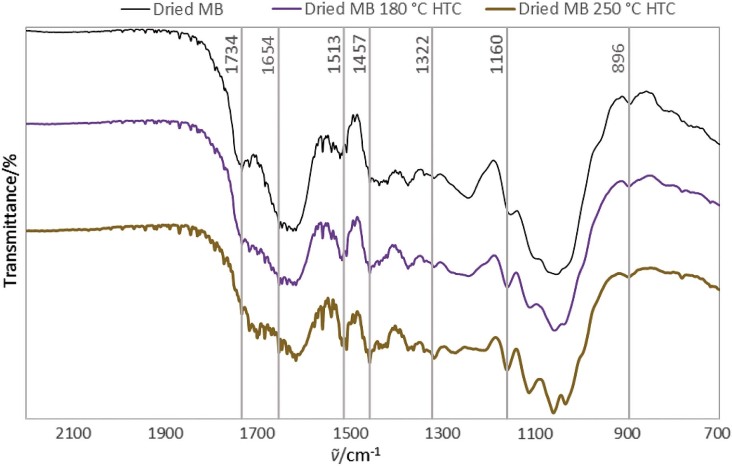
FTIR spectra of untreated dried mayhaw berry (MB) and dried MB after hydrothermal carbonization (HTC) treatments at 180 and 250 °C, showing bonds of interest

The FTIR spectra were compared in Fig. 4 for dried MB, and the solid product of this biomass after HTC treatment at either 180 or 250 °C.

Lignin vibrations appeared to become more dominant in the HTC samples: at 1322 cm^-1^ (syringyl ring and C–O stretching vibration (*31*)), 1457 cm^-1^ (CH_2_ deformation/stretching lignin and xylan (*32*)), and 1513 cm^-1^ (aromatic skeletal vibration (*33*-*36*)). Cellulose vibrations appeared to become smaller in the HTC-treated sample, particularly at 896 cm^-1^ (amorphous cellulose (*41*)) and 1160 cm^-1^ (C-O-C asymmetric stretching cellulose I and cellulose II (*39*)). These observations suggest that hemicellulose and cellulose are removed by HTC treatment, leaving the lignin that has a greater fuel value. Other researchers have reported that the concentration of lignin in HTC-treated biomass does occur (*24*). In addition, more double bonds may have been formed in condensation reactions of carbohydrate portions as evidenced in the vibration at 1654 cm^-1^ in the HTC treated spectra (*25*). The vibration for the links between hemicellulose and lignin at 1734 cm^-1^ (*37*, *38*) appeared to be smaller for the HTC-treated samples, suggesting their breakage and removal of hemicellulose.

## CONCLUSIONS

Our results indicate that the protective coating of mayhaw seeds (MS), high in lignin, prevents the hydrothermal carbonization (HTC) process from removing lower higher heating value (HHV) components from the seeds. However, the HTC process does enhance fuel value for dried mayhaw wastes (MB), with the skins (MH) and seeds (MS) unseparated. The improved HHV values means that the undried mayhaw berry wastes could be processed using HTC to densify their energy content, since water is added in the HTC process. Durable pellets were attainable from the HTC-treated biomass, as seen in friability testing. Thus, we conclude that as a by-product of food processing that is already transported, HTC-treated mayhaw jelly waste is a plausible biofuel resource.
